# Influence of testing environment and loading rate on intervertebral disc compressive mechanics: An assessment of repeatability at three different laboratories

**DOI:** 10.1002/jsp2.1110

**Published:** 2020-07-15

**Authors:** Nicolas Newell, David Rivera Tapia, Tamanna Rahman, Shiyin Lim, Grace D. O'Connell, Timothy P. Holsgrove

**Affiliations:** ^1^ Department of Mechanical Engineering Imperial College London London UK; ^2^ Department of Engineering University of Exeter Exeter UK; ^3^ Department of Mechanical Engineering University of California Berkeley California USA; ^4^ Department of Orthopaedic Surgery University of California San Francisco California USA

**Keywords:** intervertebral disc, loading rate, strain rate, test environment, test repeatability

## Abstract

In vitro mechanical testing of intervertebral discs is crucial for basic science and pre‐clinical testing. Generally, these tests aim to replicate in vivo conditions, but simplifications are necessary in specimen preparation and mechanical testing due to complexities in both structure and the loading conditions required to replicate in vivo conditions. There has been a growing interest in developing a consensus of testing protocols within the spine community to improve comparison of results between studies. The objective of this study was to perform axial compression experiments on bovine bone‐disc‐bone specimens at three institutions. No differences were observed between testing environment being air, with PBS soaked gauze, or a PBS bath (*P* > .206). A 100‐fold increase in loading rate resulted in a small (2%) but significant increase in compressive mechanics (*P* < .017). A 7% difference in compressive stiffness between Labs B and C was eliminated when values were adjusted for test system compliance. Specimens tested at Lab A, however, were found to be stiffer than specimens from Lab B and C. Even after normalizing for disc geometry and adjusting for system compliance, an ∼35% difference was observed between UK based labs (B and C) and the USA based lab (A). Large differences in specimen stiffness may be due to genetic differences between breeds or in agricultural feed and use of growth hormones; highlighting significant challenges in comparing mechanics data across studies. This research provides a standardized test protocol for the comparison of spinal specimens and provides steps towards understanding how location and test set‐up may affect biomechanical results.

## Graphical abstract

Bovine bone‐disc‐bone specimens were tested at three institutions, in three environments, and at three loading rates. No differences were observed between environments, only small differences were seen between loading rates, and although no differences were seen between the two UK based labs, a 35% difference in modulus was observed when compared to data from the USA based lab. These findings demonstrate the importance of considering these factors when comparing results obtained at different institutions.

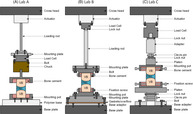

## 1 INTRODUCTION

In vitro mechanical testing of intervertebral discs (IVDs) provides a valuable tool for investigating mechanisms of disc injury and degeneration, mechanical integrity of biological repair strategies, or efficacy of medical devices. Generally, these tests aim to replicate in vivo conditions as accurately as possible, however, the complex structure and loading condition of the spine means that simplifications are necessary. As a result, there are often inconsistencies in methods used for sample preparation and testing between studies, making direct comparisons between studies a significant challenge faced by the field. There are a number of studies that have proposed standardized test methods,^1^, ^2^ primarily for testing multi‐level specimens in pure moment bending. However standard methods, specifically for axial compression tests are not available, even though physiologically the spine is subjected to axial loads and a large number of research studies limit their testing to axial compression.

Previous studies demonstrated the importance of disc hydration,^3^, ^4^, ^5^ preload,^6^, ^7^, ^8^, ^9^ and test rate.^10^, ^11^, ^12^, ^13^, ^14^, ^15^ More recently, studies have compared biomechanical testing between laboratories, to investigate the application of pure moments to multi‐level spinal specimens^16^ and to assess differences between six‐axis testing systems.^17^ These studies have highlighted the importance of consistent methods for data processing, yet there is still a lack of consistency in specimen preparation and testing conditions used throughout the spinal community.^18^ For example, differences in axial preloading, as commonly applied during spinal testing, may have substantial effects on mechanical loading outcomes^19^, ^20^ and recovery behavior.^21^ These differences make comparisons across studies challenging, or impossible.

At the 2019 Annual Meeting of the Orthopaedic Research Society (ORS), the ORS Spine Section discussed a need for consensus in biomechanical testing approaches used by the community. One challenge in developing standardized protocols is being able to determine which protocol best represents physiological loading. However, moving towards consistent test protocols, similar to ASTM International standards for testing of materials, is important for comparing data across studies. Therefore, the aim of this study was to perform axial compression experiments on bovine bone‐disc‐bone motion segments at three institutions using the same testing methods to determine whether experimental findings could be replicated across institutions, and to identify which parameters were critical for achieving comparable results to allow a move towards more standardized methods. Sample preparation and mechanical testing was performed at the University of California ‐ Berkeley (Berkeley, California), University of Exeter (Exeter, UK), and Imperial College London (London, UK), here on in referred to as Lab A, B, and C, respectively. As part of this investigation, experiments provide data regarding the effects of testing in air, wrapped in saline soaked gauze, or in a saline bath, at different strain rates.

## 2 MATERIALS AND METHODS

### 2.1 Sample preparation

Bovine IVDs have been used in this study as they have been shown to have biomechanical and biochemical similarities with nondegenerate human discs.^19^, ^22^, ^23^ Intact bovine tails were acquired from butchers or abattoirs local to each of the three institutions involved in this study, from which a total of 36 (12 at each institution) bone‐disc‐bone motion segments were acquired. Bovine tails were stored frozen at −20°C on the day of acquisition, and each tail was thawed overnight at 4°C prior to dissection and testing. Two specimens were obtained from each tail by removing soft tissue, with care taken not to damage the IVDs. The tail was then cut transversely through the first, second, and third caudal vertebral bodies at mid‐height to obtain two bone‐disc‐bone specimens. During preparation, specimens were kept hydrated through regular spraying of phosphate buffered saline (PBS, 0.15 mol/L). The width in the sagittal and coronal plane of each IVD was measured using digital calipers. The IVD height was measured using either x‐ray or micro CT, depending on equipment availability at each institution (Lab A: micro CT, Lab B: micro CT, and Lab C: x‐ray). For x‐ray measurements, a calibration stick was placed in line with the sample so that lengths could be measured. For all measurements three repeats were made and an average was calculated.

**FIGURE 1:**
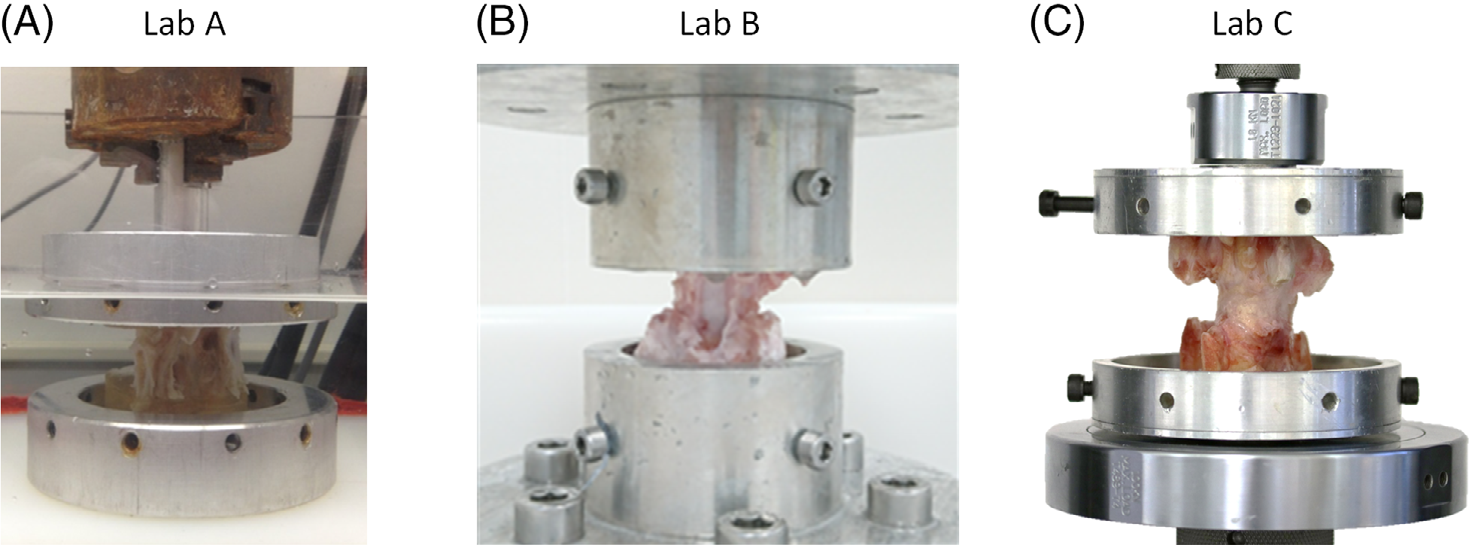
Typical specimens tested at Labs A, B, and C, with (A) showing a specimen tested in a saline bath

Each specimen was secured in polymethyl‐methacrylate (PMMA) bone cement such that the transverse plane of the IVD was horizontal and parallel with the loading platens. The specific methods to secure specimens in place differed between institutions, but broadly followed the same process of centering one vertebra within a specimen pot and fixing it in place with PMMA. The specimen was then flipped upside down to fix the other vertebral body in PMMA, while ensuring the two PMMA pots were parallel. This resulted in each institution having specimens fixed in metal pots that could be compressively loaded on testing machines (Figure 1).

### 2.2 Experimental procedure

**FIGURE 2:**
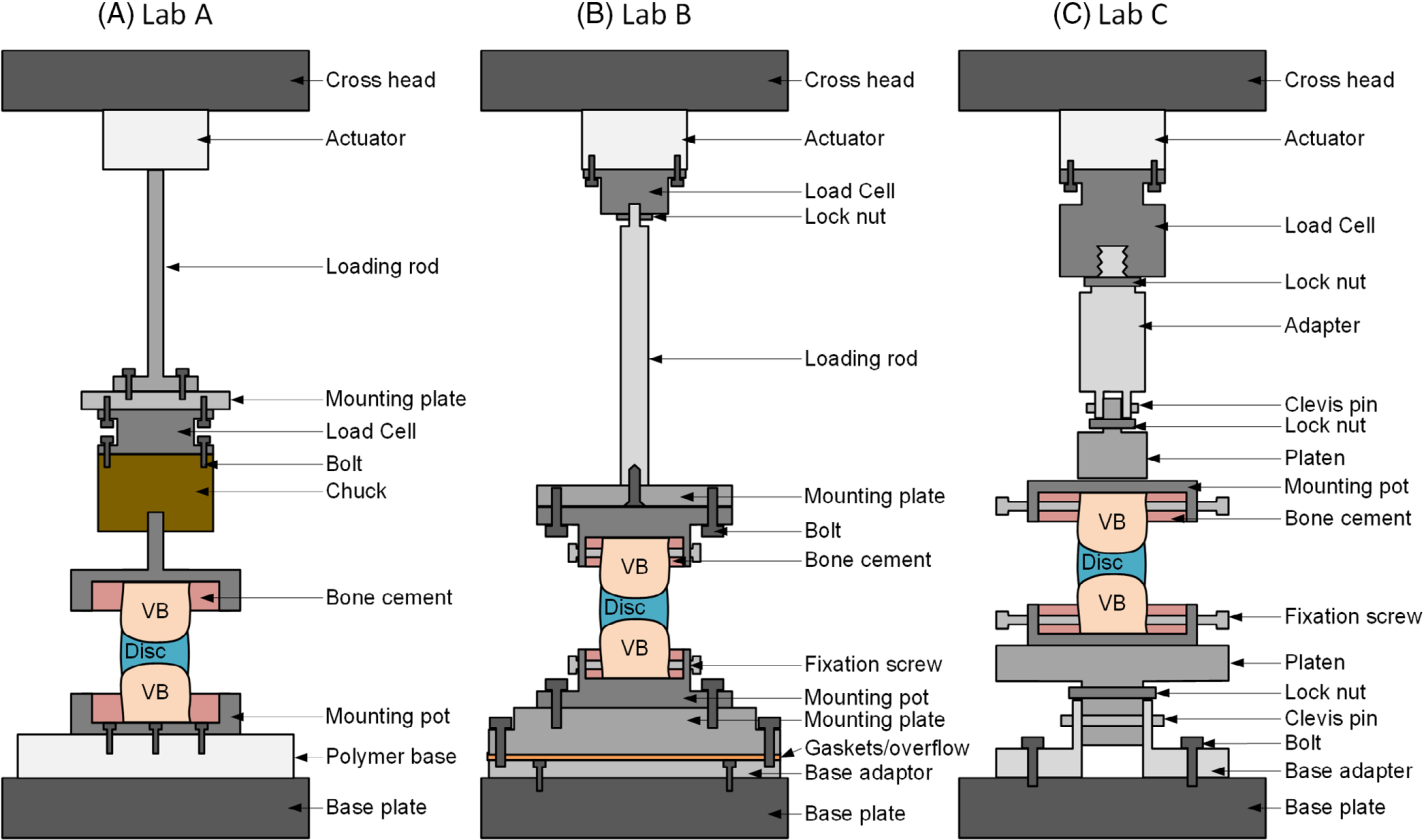
Schematic of experimental test setup and load string for a typical air test at each lab

Experiments were carried out on an MTS MiniBionix 858 (MTS Systems Corp., Eden Prairie, Minnesota), an Instron E10000 (Instron Ltd., High Wycombe, UK), and an Instron 8872 (Instron Ltd., High Wycombe, UK) at Institutions A, B, and C respectively. The load string for each of these setups in shown in Figure 2. During the process of mounting the specimens on their respective testing machines care was taken to ensure samples were not subjected to any tensile loads. Once mounted specimens were subjected to an equilibration period which involved a compressive load of 50 N for 5 minutes, followed by a 5 N compression for 15 minutes. This was followed by a compressive preload of 50 N for 5 minutes to simulate a physiological compressive force prior to the first load cycles. A 50 N compressive preload was selected to provide an initial pressure of 0.08 MPa based on previous bovine tail cross‐sectional area measurements of 622 ± 71 mm^2 24^, which is comparable to the intradiscal pressure of 0.08 to 0.11 MPa measured in‐vivo in healthy participants during various lying postures.^24^

Five cycles of axial compression were applied with a triangle wave between 50 N and 1000 N. A peak load of 1000 N was selected to provide an intradiscal pressure of ∼1.6 MPa (based on previous bovine tail cross‐sectional area measurements of 622 ± 71 mm^2 24^).^24^, ^25^ This loading was on the higher end of physiological internal pressures but low enough to ensure that damage during testing was minimal. Three loading frequencies were applied to each specimen, comprising fast (5.00 Hz), medium (0.50 Hz), and slow (0.05 Hz). To reduce the effects of repeat loading on changes in water content and losses in disc height between tests, each specimen was always tested at the fast rate first, followed by the medium rate, and finally the slow rate. Between testing at each frequency, specimens were allowed to recover with a compressive load of 5 N for 15 minutes, followed by the application of the compressive preload of 50 N for 15 minutes (Table 1).

**TABLE 1:** The test loading protocol used for each specimen at all three institutions. Shaded steps indicate the load cycles at the fast, medium and slow test frequencies

Step	Control	Data acquisition
1	Ramp to 50 N compression in 5 seconds	—
2	Hold for 5 minutes	1 Hz
3	Ramp to 5 N compression in 5 seconds	—
4	Hold for 15 minutes	1 Hz
5	Ramp to 50 N compression in 5 seconds	—
6	Hold for 5 minutes	1 Hz
7	Triangle wave between 50 N and 1000 N in compression at 5.00 Hz	1000 Hz
8	Ramp to 5 N compression in 5 seconds	—
9	Hold for 15 minutes	1 Hz
10	Ramp to 50 N compression in 5 seconds	—
11	Hold for 5 minutes	1 Hz
12	Triangle wave between 50 N and 1000 N in compression at 0.50 Hz	100 Hz
13	Ramp to 5 N compression in 5 seconds	—
14	Hold for 15 minutes	1 Hz
15	Ramp to 50 N compression in 5 seconds	—
16	Hold for 5 minutes	1 Hz
17	Triangle wave between 50 N and 1000 N in compression at 0.05 Hz	10 Hz
18	Ramp to 0 N in 5 seconds	—
End		

Previous studies report that three to five cycles is generally sufficient to obtain a repeatable force‐displacement response.^1^, ^26^ Pilot testing at each institution confirmed that force‐displacement response was repeatable after 3 cycles, with less than 2% variation in mean stiffness after the third cycle. Similarly, pilot testing was conducted to confirm that equilibration and recovery periods between testing rates allowed for sufficient disc height recovery, while still ensuring that a physiological preload (50 N compression) was maintained prior to cyclic loading.

Three variations of testing environment were investigated: air (but regularly sprayed with PBS to keep specimens hydrated); wrapped in PBS soaked gauze and food packing plastic to minimize dehydration due to evaporation; and in a PBS bath. All tests were performed at room temperature (∼22°C). Four specimens were tested in each environment at each institution, resulting in 12 tests in each environment and frequency, a total of 36 specimens, and 108 tests across all test rates (Table 2).

**TABLE 2:** Overview of testing completed across all three institutions

			Number of specimens	
Test	Test environment	Frequency (Hz)	Institution	Total
1	Air and PBS spray	5.00	4	12
2		0.50		
3		0.05		
4	Wrapped in PBS soaked gauze	5.00	4	12
5		0.50		
6		0.05		
7	PBS bath	5.00	4	12
8		0.50		
9		0.05		

In order to account for differences in clamp‐to‐clamp stiffness of each test machine, including all fixtures, a compliance test was performed at each institution with test fixtures in place. Tests were completed using five axial compressive triangle wave cycles applied between 50 N and 1000 N at 0.05 Hz.

### 2.3 Data analysis

Data was acquired at 1000 Hz for the fast rate tests, 100 Hz for the medium tests, and 10 Hz for the slow tests. Force‐displacement data from the fifth cycle was used to determine compressive stiffness, which was calculated using a linear regression between 500 and 900 N of the loading portion of the compressive cycle. This stiffness was converted to a modulus using the width and height measurements taken of each IVD. To calculate the area of the sample from the width each IVD was assumed to be circular in cross‐section.

The system compliance test was used to find a fourth power polynomial relationship of the load with respect to displacement over the compressive part of the final clamp‐to‐clamp test cycle at each institution. Using this polynomial, a compliance displacement for any load measured during the actual bovine sample experiments could be calculated and subtracted resulting in a compliance corrected force‐displacement response of the sample itself (not including any displacements of components of the test setup). No adjustments were made to the load data as this is not affected by system compliance. This method of compliance correction provided a consistent way to correct test displacement even with non‐linear system stiffness.

A 3x3 mixed factorial analysis was completed to compare the dependent variables of stiffness and compressive modulus (both uncorrected and corrected values) across the independent variables at each institution: environment (air, gauze, and bath); and test frequency (5.00, 0.50, and 0.05 Hz). Following these analyses institutions were compared using one‐way ANOVA. The specimen height, and specimen area used in the tests were compared across the three institutions using ANOVA. All tests were completed with a significance level of .05, and post‐hoc analyses were completed in cases of significance, with a Bonferroni correction used to minimize the risk of type I errors. All statistical analyses were completed in IBM SPSS Statistics software (Version 25, IBM Inc., Armonk, New York).

## 3 RESULTS

### 3.1 Specimen details

No significant differences were observed between institution in terms of disc area (mean ± SD) of 489 ± 71 mm^2^, 436 ± 92 mm^2^, and 439 ± 35 mm^2^ for Labs A, B, and C, respectively, *P* = .127), however, the disc height of specimens from Lab A (6.75 ± 0.60 mm) were approximately 20% greater than discs from Labs B (5.48 ± 1.02 mm, *P* = .033) and C (5.46 ± 1.62 mm, *P* = .029). No significant differences were observed in terms of disc area (*P* = .919) or height (*P* = .854) between the three environments.

### 3.2 Effect of environment

No significant differences were observed at any institution in either the stiffness or compressive modulus between any test environment using data before (*P* > .272 and *P* > .227, respectively) or after (*P* > .286 and *P* > .238, respectively) compliance correction. Therefore, when completing comparisons between institutions, the environment factor was pooled.

### 3.3 Effect of test rate

**FIGURE 3:**
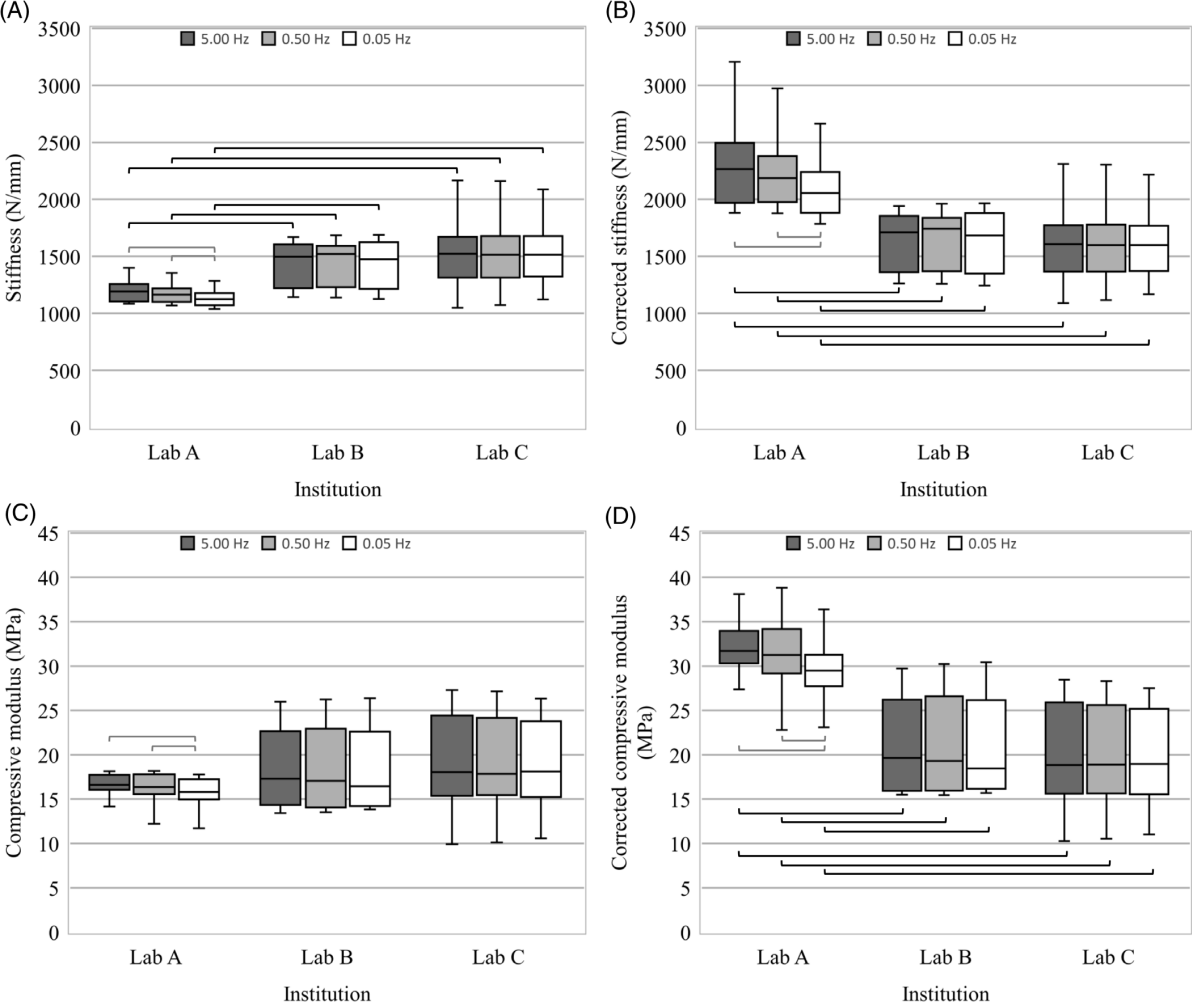
Boxplots from each institution at each test rate of the, A, uncorrected stiffness, B, corrected stiffness, C, uncorrected compressive modulus, and D corrected compressive modulus. Gray brackets indicate a significant difference (*P* < .05) in test rate identified through the mixed factorial analysis, black brackets indicate a significant difference (*P* < .05) between institutions at a given test rate identified through ANOVA

A small but significant decrease in compressive stiffness was observed between 5 Hz and 0.05 Hz (*P* < .001) and between 0.5 Hz and 0.05 Hz (*P* < .001) at Lab A. These differences were maintained after compliance correction (*P* < .007; Figure 3). Similarly, a small but significant difference was seen in compressive modulus between 5 Hz and 0.05 Hz (*P* < .001) and between 0.5 Hz and 0.05 Hz (*P* < .001), which was maintained after compliance correction (*P* < .004). Due to the significance observed between test rates at Lab A, the rate factor was not pooled in comparisons between institutions.

### 3.4 Effect of institution

As the mixed factorial analysis showed no difference between environment, this data was pooled to compare results between institution; as there was a significant difference in rate at Labs A, rate data was not pooled to compare institutions. Therefore, ANOVA were used to compare institutions at each rate for both the uncorrected and corrected stiffness and compressive moduli. These analyses showed that specimens at Lab A were significantly different to those at Labs B and C at all test rates in stiffness (*P* < .034, Figure 3A), corrected stiffness (*P* < .001, Figure 3B), and corrected compressive modulus (*P* < .002, Figure 3d). There were no significant differences between Labs B and C (*P* > .699).

Overall, the compressive stiffness of specimens from Lab A were approximately 20% lower than those at Labs B and C before compliance correction (Table 3 and Figure 3A). The compliance correction resulted in the compressive stiffness of specimens at Lab A being approximately 35% higher than at Labs B and C. There was no significant difference in the stiffness of specimens tested in Labs B and C either before or after compliance correction, but the mean stiffnesses were closer after compliance correction (Figure 3B). Compliance correction in Labs B and C increased disc joint stiffness by 5% to 14%; however, compliance correction in Lab A was greater due to a polymer base used for the PBS bath (Figure 2A). No significant differences were observed between institutions in terms of compressive modulus before (*P* > .271) compliance correction, but the compressive modulus of specimens from Lab A were significantly higher after compliance correction (*P* < .002; Figure 3c). Variation in the data increased after correcting for specimen geometry. SDs for stiffness measurements were 6.3% to 22% of the mean. In contrast, SDs for compressive modulus for each test environment was 8.5% to 40% of the mean.

**TABLE 3:** Mean ± SD compressive stiffness and compressive modulus for specimens tested in each environment at each institution at each rate

			Stiffness (N/mm)		Compressive modulus (MPa)	
Institution	Environment	Rate (Hz)	Uncorrected	Corrected	Uncorrected	Corrected
Lab A	Air	5.00	1190 ± 66	2273 ± 212	15.93 ± 1.26	30.36 ± 2.12
		0.50	1167 ± 71	2211 ± 259	15.63 ± 1.40	29.55 ± 3.14
		0.05	1125 ± 65	2065 ± 220	15.09 ± 1.50	27.64 ± 3.15
	Gauze	5.00	1209 ± 133	2373 ± 565	15.87 ± 2.55	30.92 ± 6.46
		0.50	1192 ± 119	2321 ± 466	15.65 ± 2.50	30.27 ± 5.68
		0.05	1145 ± 104	2144 ± 375	15.05 ± 2.46	28.04 ± 5.10
	Bath	5.00	1174 ± 91	2224 ± 353	18.08 ± 2.39	33.90 ± 3.01
		0.50	1161 ± 66	2180 ± 227	17.93 ± 2.64	33.46 ± 3.75
		0.05	1125 ± 61	2063 ± 204	17.38 ± 2.53	31.66 ± 3.38
Lab B	Air	5.00	1354 ± 199	1530 ± 252	19.86 ± 4.70	22.46 ± 5.68
		0.50	1372 ± 213	1554 ± 271	20.13 ± 4.87	22.82 ± 5.91
		0.05	1359 ± 211	1537 ± 269	19.96 ± 4.99	22.60 ± 6.05
	Gauze	5.00	1505 ± 201	1726 ± 259	20.18 ± 5.40	23.13 ± 6.38
		0.50	1502 ± 189	1722 ± 245	20.20 ± 5.59	23.15 ± 6.63
		0.05	1474 ± 175	1684 ± 227	19.86 ± 5.66	22.71 ± 6.71
	Bath	5.00	1409 ± 220	1601 ± 283	14.90 ± 2.24	16.86 ± 2.29
		0.50	1413 ± 242	1607 ± 311	14.92 ± 2.25	16.89 ± 2.34
		0.05	1431 ± 267	1632 ± 345	15.04 ± 1.87	17.06 ± 1.87
Lab C	Air	5.00	1333 ± 253	1399 ± 278	17.22 ± 7.80	18.10 ± 8.30
		0.50	1329 ± 238	1395 ± 262	17.17 ± 7.74	18.04 ± 8.23
		0.05	1340 ± 212	1407 ± 233	17.22 ± 7.39	18.10 ± 7.86
	Gauze	5.00	1629 ± 142	1726 ± 159	19.04 ± 4.53	19.35 ± 5.29
		0.50	1620 ± 135	1717 ± 151	18.85 ± 4.39	19.22 ± 5.10
		0.05	1606 ± 112	1701 ± 126	18.54 ± 4.38	19.04 ± 4.95
	Bath	5.00	1620 ± 390	1703 ± 433	21.02 ± 4.57	22.06 ± 4.78
		0.50	1625 ± 389	1710 ± 431	21.07 ± 4.41	22.12 ± 4.59
		0.05	1609 ± 358	1688 ± 395	20.84 ± 3.87	21.86 ± 3.99

## 4 DISCUSSION

This is the first study to investigate the axial stiffness of intervertebral discs using a combined approach to investigate the effects of institution, testing environment, and test rate. This provides novel biomechanical data and ensures that the most important factors are considered when completing tests, and when comparing previous studies that may have used different methodologies. Although similar stiffnesses and moduli were seen between the two UK based labs, significant differences were seen between these labs and the United States based lab (Lab A), even after normalizing for specimen geometry and completing a correction to account for test system compliance. The test set‐up at Lab A incorporated a polymer base beneath the specimen (Figure 2A) which accounts for the larger difference in the stiffness and modulus before and after compliance correction (Figure 3) compared to Labs B and C. Additional tests were conducted in Lab A in which specimens were tested directly on the MTS loading frame (eg, air and gauze test conditions) with the appropriate compliance correction. These tests further confirmed greater stiffness of US‐based bovine discs. Furthermore, all three labs completed compressive tests (in air at 0.05 Hz) on an ISO 10243:2010 ultra‐heavy duty die spring (diameter 50 mm, free length 102 mm), which has a known stiffness of 1215 N/mm ±10% (similar to the discs tested as part of this study). These tests demonstrated that after compliance correction all three labs measured a spring stiffness within 5.8% of the known stiffness (Lab A: 5.8%, Lab B: 0.0%, Lab C: 0.7%). This gave further confidence that the differences in disc stiffness between labs was due to the bovine samples, rather than the test setup or compliance correction method. Although the discs at all labs are expected to be from animals of roughly the same age (∼18 months), large discrepancies in disc stiffness may be due to genetic differences between different breeds of cows, differences in agriculture feed processes between countries, or differences in sex. Discrepancies in IVD mechanical properties have previously been suggested to be dependent upon breed,^1^ particularly if there are differences in internal geometries such as NP:AF ratio. Additionally, American farmers often use FDA approved steroid hormones to increase the growth rate of livestock,^27^ whilst the use of growth hormones are banned in the UK.

Had system compliance not been considered, the results would have shown that the stiffness and modulus of specimen tested at Lab A were lower, rather than higher than specimens tested at Labs B and C, as was the case after compliance correction. This highlights the importance of accounting for system compliance, either through compliance correction methods, as used in the present study, by using a decoupled displacement measurement system,^10^, ^13^, ^28^ or a contactless measurement system with appropriate accuracy for the relatively small displacements that occur during axial testing of the IVD.^29^ Two commonly used contactless systems are digital image correlation (DIC) with speckle coating and marker‐based motion capture which inherently account for system compliance. DIC however is generally limited to tests conducted in air due to issues with calibrating through fluids, and the lights required for high rate data capture can have a heating effect on specimens. Marker‐based motion capture systems generally have accuracy in the region of 0.1 mm which is not sufficient for all biomechanical studies. Importantly, whether data has been adjusted for machine compliance should be considered when comparing results between studies, and where possible materials that have a significantly higher modulus than the specimen being tested should be used in the load string to reduce system compliance.

Testing specimens in air, wrapped in gauze, or in a PBS bath did not significantly affect the stiffness or modulus of the specimens analyzed in this study. Conversely, Costi et al^4^ reported a 20% to 30% decrease in ovine IVD stiffness in torsion, axial compression and lateral bending when testing in a bath. Race et al^15^ also reported a decrease in bovine IVD stiffness after 30 minutes of creep‐induced dehydration. The difference in these findings compared to the present study could be due to several reasons. Firstly, the soaking time used in this study was limited to 20 minutes to prevent over‐hydration, but this soak time was shorter than soak times in the literature (3‐4 hours in Costi et al^4^ and up to 8 hours in Race et al^15^). Bezci et al^30^ observed a 35% decrease in axial compressive stiffness when water absorption by the disc was restricted through osmotic loading. Thus, it is likely that specimens in this study did not absorb a sufficient amount of water during the 20‐minute soak to alter disc stiffness. Secondly, PBS bath tests were conducted at room temperature (∼22°C) compared to 37°C in Costi et al^4^ The effect of temperature on elastic mechanics is not clear with conflicting data in the literature. Previous studies have shown no differences in elastic mechanics with temperature,^31^, ^32^ but Huang et al^32^ observed a nonlinear increase in viscous mechanical behavior with temperature. Therefore, environmental or rate dependent effects may have been more pronounced in this study if the PBS bath was at 37°C rather than room temperature.

As with previous investigations^13^, ^14^, ^15^, ^33^, ^34^ a statistically significant increase in stiffness was seen with testing rate, but only at Lab A. However, in agreement with human in vitro studies, this increase was small in comparison to interspecimen variation.^13^ Although 1 Hz is often cited for physiological loading purposes, this finding suggests that faster loading rates may be able to be applied to reduce testing time, also potentially reducing the need to test in a saline bath.

The mechanical behavior of bovine and human IVDs has been found to be similar,^23^ suggesting that disc tissue properties may also be comparable. However, care must be taken when interpreting the environment and rate related results presented in this study, as we evaluated disc mechanics of healthy bovine discs. Changes in disc composition with degeneration may have a greater impact on disc mechanics with respect to test environment and rate. Labs B and C were both located in the same country (UK), and the compressive stiffness from these labs were not significantly different from one another but were different to Lab A (United States). This highlights that despite using different testing systems, and acquiring specimens from different sources, the UK labs resulted in highly comparable stiffness values. Though this does not confirm the reasons for the large differences observed between Lab A and Labs B and C, it does suggest that there is value in completing further research into the effects of animal breed and husbandry on the biomechanical properties of spinal specimens.

The compressive loads of 50‐1000 N used for the present study were selected to cause intradiscal pressures that range from a lying posture to high physiological loading (intradiscal pressure range: 0.08‐1.6 MPa).^24^ However, because the mean cross‐sectional area of 455 mm^2^ for the specimens in the present study was less than the 622 mm^2^ ^35^ previously reported in the literature, the pressures applied to the specimens was higher than planned. However, the pressure at 50 N, which is expected to be related to an intradiscal pressure of 0.11 MPa, which is still comparable to the intradiscal pressure during lying postures, and the pressure at 1000 N would be 2.2 MPa, which is quite high. However, it should be noted that the conversion between applied load and intradiscal pressure were extrapolated from coupling in vitro and in vivo data from human discs, and may not directly apply to bovine discs.^24^, ^25^

In conclusion, although the test rate can have an effect on the compressive properties, this is small compared to the interspecimen and inter‐laboratory variability. Even after accounting for specimen dimensions and clamp‐to‐clamp compliance, compressive mechanical properties were found to be different between institutions, which may be due to the variability of specimens local to different research laboratories. Care must therefore be taken to consider this when comparing results between studies and the following recommendations are suggested to move towards more standardized results between labs:

1. Any compliance in the load string should be reported and accounted for in post‐test analysis to ensure only IVD displacements are assessed.
2. For tests shorter than 90 minutes, testing environment and rate does not make a large difference. Therefore, we suggest wrapping specimens in in PBS soaked gauze during testing to reduce the need for additional fixtures (eg, water bath) and to prevent the IVD from becoming dehydrated, as would be the case when testing in air. Tests only need to be conducted at a single rate; we recommend 0.5 Hz or ∼1 mm/s as they are closest to physiological rates that most testing machines can achieve.
3. If possible, the breed and species of the specimens should be reported to provide more information regarding this effect in the future.

## CONFLICT OF INTEREST

The authors have no conflicts of interest to declare.

## AUTHOR CONTRIBUTIONS

Nicolas Newel, Grace D. O'Connell, and Timothy P. Holsgrove designed the research. David Rivera Tapia, Tamanna Rahman, and Shiyin Lim performed the experiments. Nicolas Newel, David Rivera Tapia, Tamanna Rahman, Shiyin Lim, Grace D. O'Connell, and Timothy P. Holsgrove analyzed and interpreted data. Nicolas Newel, Grace D. O'Connell, and Timothy P. Holsgrove wrote the manuscript and all authors revised.
